# The ethics of anti-love drugs qua precommitment strategy

**DOI:** 10.1007/s11017-025-09721-6

**Published:** 2025-07-05

**Authors:** Bernard Long

**Affiliations:** School of Law, University College Cork, Cork, Ireland

**Keywords:** Anti-love drugs, Love drugs, Precommitment, Autonomy

## Abstract

The ethics of anti-love drugs – pharmaceutical interventions to dampen one’s feelings of love for, say, a former partner – have been the subject of a growing body of research. Scientific research on these drugs is fairly nascent and ethical debates about their implications are therefore by necessity largely speculative. Nonetheless, insofar as future developments in anti-love drugs propose to affect a value as personal and important as love, these ethical debates are imperative. In this article, I propose to add a new dimension to ethical discourse on anti-love drugs by contextualising it within existing ethical debates on precommitment. An agent who consumes an anti-love drug does so to limit their future behavior – i.e. preventing themselves from reigniting their former relationship—based on their present preferences. The use of anti-love drugs is therefore an unambiguous example of a precommitment strategy. This recognition therefore allows one to draw on existing ethical research on precommitment to invigorate ethical discourse on anti-love drugs.

## Introduction

In his book, *Don’t Text Your Ex Happy Birthday*, Nick Viall, a former star of *The Bachelor*, exhorts the reader to resist the temptation to contact their erstwhile romantic partner [1]. While one might advise others that it would be foolish to rekindle their relationship, Viall says, our own waning relationships are treated with an attitude of "If we truly love each other then we can get through anything" [1, p. 187]. The double standard Viall identifies is one which I assume is familiar to many. After a traumatic incident (such as infidelity or abuse), a series of explosive arguments or merely a long period of boredom and/or glaring incompatibility, one might understandably decide to terminate their relationship and move on to greener pastures. As wise and straightforward as this conclusion might seem in the moment, however, one’s resolve is wont to waver when confronted with long and lonely nights spent yearning, the fading of bad memories and increased salience of good ones, or even the realization that other prospective partners lack the qualities that made one’s previous partner so appealing. The temptation to renege on one’s resolution to break up may become irresistible.

The search for a solution to this quandary – a way to overcome feelings of romantic attachment and attraction; in other words, a ‘cure for love’ – has received attention since antiquity. Ovid, for instance, outlines a series of methods "to extinguish the fire that burns in your breast and to free the heart that has been enslaved" [2]. These methods include distractive labor, reflection on the wrongs committed by one’s lover, and the seeking out of a different sexual partner. Though written over two millennia later, Viall’s advice (including exercise and reflection) is remarkably similar, though he diverges from Ovid in recommending not to rush back into dating following a break-up [1]. In this article, I examine a pharmaceutical cure for love, so-called “anti-love drugs” [3], which “manipulate brain systems at whim to…diminish our love for one another” [4].^1^ The ethics of pharmaceutical interventions to either preserve and bolster love (love drugs) or quell it (anti-love drugs) continues to be debated in a growing body of literature [3, 5–12]. Here, I aim to contribute to the ethical discourse surrounding anti-love drugs by contextualizing them within existing ethical debates on precommitment. While I will outline precommitment in more detail later, precommitment strategies involve self-imposed constraints [13], the purpose of which is to ensure that one’s future behavior will adhere to one’s present preference about what that future behavior should be [14].^2^ The place of anti-love drugs within this definition is clear. A person who has resolved to end and move on from their relationship knows that their future pangs of love may cause them to renege on this resolution and reignite their relationship. An anti-love drug operates to preclude this backsliding (or at least make it less likely) by dampening a person’s feelings of love, thereby ensuring that their future behavior conforms to their present preference to break up and move on.^3^

Recognizing anti-love drugs as a precommitment strategy^4^ enlivens debates about their ethics by situating them within a broader and well-developed discursive framework. I will begin by briefly outlining the objects of discussion (love, anti-love drugs, and precommitment). Next, I will sketch the specific challenge anti-love drugs *qua* precommitment strategy seek to address. Specifically, I will offer a conceptualization of the desire to return to a former partner as a form of *akrasia* – generally translated as a lack of self-control, weakness of the will or incontinence – and demonstrate the role of precommitment strategies, including anti-love drugs, in addressing this problem. Finally, drawing on existing ethical arguments regarding precommitment, I will engage with the normative permissibility of anti-love drugs. Ultimately, I will conclude that, while anti-love drugs are not *prima facie* objectionable, the fundamentally subjective and personal dimension of love ought to circumscribe their availability.

## The parameters of the present discussion

Before proceeding to the more substantive aspects of this article, it is first necessary to briefly outline some core concepts. The first is to define the main object of discussion: anti-love drugs. Defining drugs is, according to Benedetti, “an easy task” [17]. He writes,

[A drug] is a molecule delivered to the body to produce a biological effect. Its mode of action is to alter one or more biochemical pathways, for instance, by binding to a receptor or by modifying the activity of an enzyme. [17, p. 239]

While Benedetti’s belief in the apparent ease of defining drugs and, with it, his own definition have been criticized [18], it is, I believe, sufficient to adopt for present purposes. Much current work in the field of love and anti-love drugs (or, more broadly, biotechnology) is admittedly speculative [6].^5^ Nonetheless, the speculative nature of certain aspects of bioethics does not render their discussion futile. As Decamp and Buchanan argue, pre-emptive ethical discussion “may help *shape* the technological development” of biochemical advancements [19]. Moreover, they opine that it may be “more prudent to explore a range of possible issues, some of which may not arise, than to be overtaken by events owing to the failure to think ahead” [19]. Finally, Earp and colleagues comment that “reflecting on plausible ‘future’ outcomes can help to clarify and simplify the underlying moral equations involved”, thereby contributing to ethical discourse on analogous, extant cases [6]. In the absence of an extant biotechnological or biomedical intervention to quell each aspect of romantic feelings – lust, attraction and attachment [20] – towards *one* person,^6^ a speculative, though rebuttable, model is necessary. It might, of course, be the case that anti-love biotechnology will take the form of biochips [6] or a cocktail of drugs, rather than a single pill. For present purposes, I assume the future availability of a single drug which, per Benedetti’s [17] definition, alters one’s biochemical pathways to dampen or eradicate feelings of love towards a specific person, such as an erstwhile romantic partner.^7^

Though implicit from the foregoing, it is also necessary to make explicit this article’s emphasis on love’s biological basis. Love has a dual nature [3, 22]. Love’s biological aspect, which I consider in this article, refers to the “set of overlapping but functionally distinct brain systems that evolved to suit the reproductive needs of our ancestors” [3]. Operating parallel to these biological factors, however, is the societal, social/psychosocial, cultural and historical element to love [3, 22, 23]. This aspect acknowledges that, as Diamond observes, “human experiences of sexual arousal and romantic love are always mediated by social, cultural, and interpersonal contexts” [23]. I do not wish, of course, to disparage the importance of the social aspect to love.^8^ Indeed, a person might even enact a socially-oriented precommitment strategy against reigniting a former relationship. They might, for instance, mimic a cocaine addict from one popular country song by instructing a family member to lock them in a room until their desire for their vice (in this case, their former partner) has subsided [16]. In the sense that this article considers the ethics of biochemical pathway-altering drugs, however, its focus is unambiguously on the biological aspect of love.

Next, because this article deals with anti-love drugs, it is important to define what is meant by love. Here, I adopt a subjective view, identifying love with a person’s subjective experience thereof and feelings thereabout. Earp and Savulescu reject what they call a “medicalized” conception of love, which only considers love to count if it is healthy [3, pp. 10–11]. The tendency to adopt a normative conception of love is, of course, entirely understandable. Love is a notion which is valorized and insisting that it only applies to normatively desirable scenarios preserves it from being tarnished by association with harmful or destructive scenarios. I do not, however, intend to associate love solely with objective values of care, stability, good communication, and so on. Rather, I follow Brogaard in considering that love is an emotion and can therefore be either rational or irrational [24]. As such, the feelings which draw a person towards a former relationship count as love whether that relationship is healthy or unhealthy, helpful or harmful. The subjective perspective on love which I adopt is also essential insofar as it interacts with another concept which is central to this article: *akrasia*. An *akratēs* is a person who reneges on their resolutions [25]. Crucially, however, the resolution they override is one which is *self*-judged as normatively desirable, rather than being correct in some objective sense. My position is therefore clear; both the resolution to leave a relationship and the love which draws a person back to it are to be assessed relative to that person’s subjective preferences and emotions.

Finally, a core aspect of this article is precommitment strategies, whereby a person manipulates the circumstances of their future decision-making to ensure that their future behavior aligns with their current preference thereabout. There is, however, a crucial distinction between precommitment devices which affect the future options or behavior of agents who either possess or lack capacity, respectively, at the future time [16]. Advance healthcare directives (AHDs) are an example of the latter; they govern the future treatment of a person at a time when they will lack decision-making capacity. Gambling self-exclusion agreements, in contrast, affect the future options of a person who will possess decision-making capacity at the future time and may express a preference contrary to their initial resolution. Anti-love drugs are, of course, more closely analogized to self-exclusion agreements than they are to AHDs. An erstwhile lover is not, of course, lacking capacity in the way a person with dementia or in a persistent vegetative state is. Notwithstanding the effect of the drug on their preference formation, which I will consider later, they are entirely at leisure to develop and express a preference contrary to their resolution to end the relationship; indeed, the point of anti-love drugs is to address this contrary preference.

## Akratic love

While I have thus far characterized the desire to return to a former relationship as one which is motivated by love, this characterization is insufficient. Love, of course, motivates all manner of actions, including entering a relationship, staying in a relationship, and sacrificing oneself for one’s partner. What is needed is a theoretical model which explains the specific phenomenon of desiring to return to a former partner. Here I intend to characterize this desire as a form of *akrasia*, a concept with roots in antiquity and which has attracted a surge of renewed attention since the mid-twentieth century [27]. *Akrasia* is, as I have said, variously translated as weakness of the will, a lack of self-control and incontinence [25, 27–30]. Despite varying translations, the character trait *akrasia* describes is clear. As Aristotle writes, “the continent man abides by his resolutions more and the incontinent man less than most men can” [25]. The *akratēs*, simply put, is a person who can rationally identify, say, the harmfulness of a particular activity (such as gambling, unhealthy eating, or returning to a former partner), acknowledge the worthwhileness of abstaining therefrom, and resolve to abstain in future. However, whereas some may possess sufficient willpower to render this resolution effective on its own merits, the *akratēs* does not. Rather, their willpower is apt to subside, their resolution to be discarded and the behavior which they resolved to abjure to instead be embraced. The akratic erstwhile lover, then, is a person who can judge their relationship to be somehow unsatisfactory and resolve to end it. The wavering of their willpower, however, means that they cannot cleave to this resolution and instead are likely to abjure it and return to their former partner.

An important point which must be made about *akrasia* is that it differs from a simple change of mind, though these respective phenomena are similar. As regards a non-akratic change of mind, imagine a person waiting to order at a restaurant who changes their mind at the last minute from a margherita pizza to a *quattro formaggi*. Though the restaurant patron’s initial intention has given way to a preference reversal, they cannot be said to be akratic, especially in the sense that *akrasia* can be equated with weakness of the will. Rather, *akrasia* relates to the abandonment of what Holton refers to as a “special sort of intention” – one which is supposed to withstand foreseen contrary desires – which he calls a resolution [31, p. 42]. To illustrate the difference between an ordinary intention and a resolution, then, one might return to the indecisive restaurant patron. While the patron’s sudden preference for *quattro formaggi* pizza is non-akratic, one might imagine that their companion goes to the restaurant having intended to eat healthily and therefore plans to order a salad. At the last minute, however, they suffer a bout of *akrasia*, abandon their healthy-eating resolution and order a calorie-dense burger. This preference reversal is undoubtedly akratic. While the difference between a margherita pizza and a *quattro formaggi* is essentially trifling, the choice of a salad instead of a burger, on the other hand, represents the “special sort of intention” of which Holton writes and to renege on this intention at the last moment is quintessentially akratic. Assume, for now, that the desire to terminate a relationship *does* (or at least can) represent a “special sort of intention” or resolution, which is intended to withstand the contrary desire to reignite it. While I will acknowledge later that the termination of a relationship may be flippant or subjectively inferior to the decision to stay together, a romantic relationship can represent one of the most formative and significant experiences in one’s life, as can the decision to end one. Choices about whether to end or return to a relationship therefore carry significantly more gravitas than the choice of pizza toppings.

The notion that an akratic agent acts against their own judgment of the good is one which opponents of *akrasia* deny as a logical impossibility [32]. This position insists that no one goes willingly towards evil (which is the very presupposition upon which *akrasia* presumably relies) based on the assumption that when an agent acts on a desire, this action is inexorable from the value judgements inherent in that desire [33]. There is, of course, some merit to this assumption. From a third-person perspective, at least, a person returning to a former partner does seem to imply that they love them and, on some level, *want* to be in a relationship with them. Despite this ostensible merit, however, it ultimately relies on the assumption that all judgement-desire-action compounds are essentially qualitatively equal. In contrast, Segvic remarks that proponents of *akrasia* typically consider “the so-called 'better judgement' as something more than a mere judgement that some course of action is better" [33, p. 74]. She goes on, “The 'better judgement' is 'better' because it is reflectively endorsed; or because it has higher epistemic credentials; or because it is the judgement with which the person more fully and directly identifies" [33]. Accepting this position entails identifying some judgements, to borrow the language of autonomy, as first-order judgements and others as superior second-order or higher-order judgements [34, 35]. First-order judgements need not, of course, be severed from desires and their inherent value judgements; *akrasia* need not assume that an agent acts randomly and for no reason at all. However, this position accepts that second-order judgements are qualitatively superior.

A more perspicuous model for conceptualizing the desire to return to a former partner as *akrasia* is found in Heather and Segal’s explanation of relapse by addicts as *akrasia* [36]. Using the example of a smoker trying to quit, they acknowledge that the smoker, as deniers of *akrasia* contend, has a reason (*r*) to relapse, namely that it will bring immediate enjoyment. Knowing that immediate enjoyment is not the only relevant interest, however, the smoker has a more comprehensive reason (*r’*) not to relapse. Here, *r’*, being a reason based on all the information presently available to the smoker, includes *r*. If the smoker relapses, this behavior is akratic because it is motivated by an insufficient reason, *r*, in comparison to the overall better reason, *r’*, which subsumes *r*. Conceptualizing *akrasia* in this manner intelligizes the desire to return to a former relationship, and provides a vital answer to the objections of *akrasia*’s opponents. Heather and Segal’s model acknowledges that the erstwhile lover’s return is not detached from the usual relationship between value judgments, desires, and actions. Instead, it affirms this action as akratic by outlining how this action is altogether inferior to the agent’s superior, all-things-considered judgement.

Though the main purpose of this article is to contextualize anti-love drugs within existing ethical debates on precommitment, conceptualizing the desire to return as ‘akratic love’ also refutes one argument against love and anti-love drugs. The gist of this argument is that biochemically enhancing or diminishing love – or pharmaceutical alteration of human cognition and emotion, generally – may be normatively objectionable because the feelings produced are inauthentic [12, 37, 38]. This position is adroitly summarised by the President’s Council on Bioethics, in the context of memory-blunting drugs, who write of the danger of “falsifying our perception of the world and undermining our true identity” [37].

The notion that drugs may undermine one’s authentic identity in the name of securing happiness or some similar value is one with distinctly dystopian undertones. In Aldous Huxley’s *magnum opus*, *Brave New World*, for instance, the populace is pacified by soma, a pleasant, euphoria-inducing, hallucinogenic drug which provides its user with a “holiday from reality” [39]. Though I do not wish to diminish the significance of this threat in a general sense, I do not consider it relevant here. Anti-love drugs, as I have outlined, are intended to address an agent’s *akrasia*, namely their desire to return to a relationship they have resolved to end. Nonetheless, the account of *akrasia* which I have advanced frames a person’s akratic desire as a lower-order preference with inferior reasons to their superiorly-reasoned, higher-order, ‘better’ judgement.^9^ Crucially, however, each of these contradictory preferences arises from the subjective will of the agent; neither is inauthentically alien and imposed only by drug consumption. Anti-love drugs therefore only represent the biochemical enhancement of an agent’s existing, subjective resolution relative to their contrary preference to backslide, rather than the pharmaceutical introduction of an alien and inauthentic desire to break up.

## Precommitment as a solution to akratic love

Acknowledging that the erstwhile lover is an *akratēs* presents a significant dilemma. Despite knowing that remaining apart from their former partner is the self-adjudged best course of action, their willpower is insufficiently strong to reliably ensure that they will act accordingly. How, then, do anti-love drugs resolve this dilemma? In the context of addiction,^10^ Elster writes that precommitment strategies represent a logical solution to the problem of weakness of the will by providing “a technique for quitting that does not rely merely on [the] will” [13]. Precommitment, as I have said, involves the self-imposition of constraints to ensure that one’s future behavior will conform to one’s present preferences about what it ought to be. In other words, a precommitting agent forms a preference at one point in time (*t*^*1*^) about what their behavior at a future point (*t*^*2*^) should be (this may involve either the performance or non-performance of some act). In order to ensure that their *t*^*2*^ behavior conforms to their *t*^*1*^ preference thereabout, they enact a barrier to their ability to act contrary to their *t*^*1*^ preference at *t*^*2*^. In short, precommitment involves the manipulation of the circumstances of one’s future decision-making to ensure that future behavior aligns with one’s current preference thereabout. The solution precommitment provides for akratic backsliding is clear. Foreseeing at *t*^*1*^ that their *akrasia* may cause their lower-order, inferior desire to prevail over their better judgement at *t*^*2*^, a person may enact a precommitment strategy at *t*^*1*^ to limit or preclude their ability to act on their lesser desire at *t*^*2*^ and thereby ensure that their *t*^*1*^ preference instead prevails.

Precommitment encompasses a diverse array of strategies. Most straightforwardly, these include Ulysses pacts, where a person relinquishes access to their vice to another party and implores them to withhold access when the precommitting person experiences a preference reversal at *t*^*2*^ [15]. The archetypal example of a Ulysses pact is the eponymous agreement between Ulysses and his sailors. Ulysses, eager to hear the Sirens’ song but cognizant of the ruination that might accompany it, fills the ears of the sailors under his command with wax, temporarily deafening them, and instructs them to tie him to the ship’s mast and not release him even if he later orders them to. Upon hearing the Sirens’ enticements, Ulysses is overcome with temptation and protests to his men to release him but, relying on his prior instruction, they do not. Other examples include gambling self-exclusion agreements and, as I mentioned previously, having a family member or friend lock oneself in a room until the urge for relapse has subsided [16]. Precommitment is not limited to the complete removal of options, as in Ulysses pacts, however. Another category – the imposition of costs for acting contrary to one’s *t*^*1*^ preference – neatly brings the discussion around to biochemical, drug-induced precommitment. Disulfiram, for example, is used in the treatment of alcohol use disorder. It discourages alcohol consumption by producing acute ethanol sensitivity and inducing negative physiological responses when alcohol is ingested. To be sure, consuming disulfiram does not remove the option of relapse for a recovering alcoholic; alcohol remains readily available and they may consume it at will. Ingesting disulfiram does, however, increase the likelihood that a recovering alcoholic’s *t*^*2*^ behavior will conform to their presumable *t*^*1*^ preference to abstain from alcohol by necessarily associating alcohol consumption with negative physiological effects.

On one hand, anti-love drugs operate in a way which is distinct from Ulysses pacts or the imposition of costs. Relative to the formation of an akratic *t*^*2*^ preference, Ulysses pacts and disulfiram, for example, operate in a sort of *ex post facto* manner to prevent an agent acting (or at least reduce their likelihood of acting) upon a *t*^*2*^ preference which they have already formed. Ulysses’ pact with his sailors does not prevent him from desiring to pursue the Sirens at *t*^*2*^; it merely prevents him from acting upon this desire. Similarly, disulfiram does not reduce cravings for alcohol and therefore does not prevent a recovering alcoholic from developing a *t*^*2*^ preference for relapse. It does, however, decrease the likelihood that they will act upon this preference by imposing inexorable costs on doing so. In contrast, by affecting biochemical pathways to limit feelings of love, anti-love drugs operate in an *ex ante* fashion to prevent the formation of an undesired *t*^*2*^ preference at all.^11^ This distinction seems to imply that anti-love drugs ought to be treated as ethically distinct from *ex post facto* forms of precommitment. In truth, however, anti-love drugs’ *ex ante* operation only raises the question of authenticity I earlier dispelled. Whereas Ulysses experiences a clearly authentic (though epistemically inferior) *t*^*2*^ desire to contravene his *t*^*1*^ preference, a person under the influence of anti-love drugs might be thought to inauthentically fail to develop an equivalent preference to return to their former partner. In fact, however, this failure to develop a contrary preference is not inauthentic because it coheres with their authentic *t*^*1*^ preference to end the relationship.^12^ Putting this distinction to one side, then, it is clear that anti-love drugs fit into the definition of precommitment I outlined earlier. By consuming an anti-love drug at *t*^*1*^, a person manipulates the circumstances of their future decision-making to ensure that their *t*^*2*^ behavior remains consistent with their *t*^*1*^ desire to end their relationship and not return thereto.

## Objections to precommitment

Acknowledging that anti-love drugs constitute a precommitment strategy to address akratic love adds a vital dimension to discourse around their ethics by contextualizing this discourse within an existing discursive framework on the normativity of precommitment. In this section, I will examine some objections to precommitment and argue that there are no knockdown reasons for objecting to precommitment, which indicates that there are similarly no *prima facie* reasons to object to anti-love drugs.

In assessing the normative permissibility of precommitment, the fundamental question is whether it is ethically acceptable to bind one’s future self in a way that one’s future self rejects (or, as is the case with anti-love drugs, *would* have rejected). Brock is one theorist who objects to precommitment, which he characterizes as a “tyranny of the present” [43, p. 1810]. Viewing precommitment through the prism of autonomy, he writes that, inasmuch as being autonomous means to make choices for one’s life according to one’s own conception of the good life and free from interference by others, it also involves the ability to revise one’s conception of the good life and according life plans, and to put these revised plans into effect. Precommitment strategies, he argues, unduly limit the future self’s ability to exercize autonomy in this way and are therefore – in cases where there is no defect in the agent’s *t*^*2*^ decision-making^13^ – impermissible. One might call this the ‘autonomy objection.’

I reject the autonomy objection along two interrelated lines. First, though it is impossible to properly engage with the vast and complex body of literature on autonomy here, personal autonomy is, in simple terms, associated with “steering the direction of one’s life, determining how to behave, and deciding what projects to engage in” [44] and being the “helmsman” of one’s own life and actions [45]. Insofar as *akrasia* renders it impossible for an agent to cleave to a project they have autonomously chosen to engage in, it prevents them from helming or steering their own life and is therefore inimical to autonomy. Enacting a precommitment strategy, on the other hand, represents an autonomous act on the part of the agent to address their own *akrasia*; it allows them to helm and steer their life by manipulating their own decision-making circumstances to ensure that they cleave to the projects they have autonomously endorsed. Insofar as precommitment offers a self-driven solution to the problem of *akrasia*, then, it bolsters an agent’s autonomy, rather than diminishes it.^14^ In short, while allowing precommitment may permit a tyranny of the present, prohibiting it would impugn autonomy and promote an anarchy of the self.^15^ Secondly, while Brock is concerned that precommitment limits future autonomy, it is a simple fact that many (perhaps all) autonomous, present decisions preclude certain future exercises of autonomy. I might, for instance, choose (owing to cowardice) not to seize the moment and ask the object of my desire on a date. Assuming that this fetching person will not wait forever for me to make my move, the moment may pass and my choice not to act forever forecloses my future autonomy to make them breakfast, buy them a Valentine’s gift, or ask them to marry me. The autonomy objection therefore fails because there is, in fact, nothing unique about precommitment’s limitation of future autonomy.

An alternative objection to precommitment comes from Mill. In *Principles of Political Economy*, Mill rejected,

when an individual attempt [is made] to decide irrevocably now, what will be best for his interest at some future and distant time. The presumption in favour of individual judgment is only legitimate where the judgment is grounded on actual, and especially on present, personal experience; not where it is formed antecedently to experience, and not suffered to be reversed even after experience has condemned it [46].

Restating Mill’s objection in the context of anti-love drugs, it is true in theory, though not in practice, that the *t*^*2*^ agent who wishes to reignite a former relationship is better-informed than they were when they formed a *t*^*1*^ preference to remain apart. At *t*^*1*^, the person’s preference is based on some degree of personal experience, presumably that their relationship is abusive, boring, or otherwise unsatisfactory. They cannot, however, *know* quite how much they will pine for their partner or how empty their life will feel without them. At *t*^*2*^, however, the agent theoretically has access to all of the information which motivated their *t*^*1*^ decision *and* all subsequent information, namely how much they miss their former partner. Precommitment is normatively undesirable, Mill’s argument goes, because it allows a poorly-informed present self to bind a better-informed future self. I will refer to this as the ‘greater information objection.’

Though enjoying some ostensible theoretical merit, however, the greater information objection is ultimately grounded on the fallacious assumption of a rational agent whose decision-making capacity increases in a linear manner as they consume and incorporate information. There are, however, strong reasons to reject this assumption. For one, recency bias, whereby information and events which occurred more recently are more easily recalled and disproportionately valued, indicates that agents may inappropriately prioritize information acquired more recently [47–49]. In the case of the erstwhile lover, while the information available at *t*^*2*^ is theoretically greater than that available at *t*^*1*^, it is entirely likely that their incorporation of this greater information is unbalanced and irrational. The more recent feelings of pining and loss may be sharper and therefore more salient than the feelings which induced the person to end the relationship in the first place. In a similar vein, *akrasia*, the very problem which precommitment seeks to resolve, demonstrates that an agent’s decision-making does not increase linearly in rationality. Rather, the *akratēs* is wont to momentarily act according to their lesser preferences and eschew their prior superior judgement. Acknowledging, then, that the *t*^*2*^ agent is not necessarily better-informed or, at least, better able to act rationally according to their greater knowledge, it becomes apparent that Mill’s opposition to precommitment is misplaced.

In this section, I have presented two objections to precommitment. Relying on Brock’s and Mill’s respective accounts as exemplars, I outlined the autonomy objection and the greater information objection, and argued that each of these is misplaced. Recognizing that neither of these approaches provides a *prima facie* reason to reject precommitment, it must therefore be accepted that precommitment (including anti-love drugs) is normatively permissible in at least some circumstances.

## Circumscribing anti-love drugs’ availability

Thus far, I have assumed a somewhat straightforward scenario. I have envisioned a person who has ended a relationship (as a superior, higher-order preference) and now wishes to return thereto (as an akratic, lower-order preference). In truth, however, determining whether the decision to end a relationship is the higher-order one and the desire to return the lower-order is an inexact science. The apparently akratic agent is, of course, an unreliable narrator of their own preferences, while whether a preference belongs to a higher or lower order is largely inscrutable to third parties [16]. Insofar as precommitment often protects an agent from some harmful vice, such as gambling to excess or a toxic relationship, it is tempting to consider the self-binding decision to represent a higher-order preference which restricts the later, lower-order preference. This, however, is an assumption which is not justified in all cases. Elster gives the example of covenant marriage, a form of marriage which is more difficult to enter and dissolve than ordinary marriages [13]. It is easy to envision how an engaged person might be motivated by a putatively ‘inferior’ reason to opt for a covenant marriage, such as intense infatuation or a reluctance to imply to their prospective spouse that their commitment is not absolute. This decision, however, might bind a later, ‘superior’ decision to divorce – grounded in self-respect or the value of marital faithfulness—when the person’s spouse commits infidelity.

To return to the main theme of this article, imagine a person (Brett) who has a generally healthy and fulfilling relationship with his partner (Tiffany). Imagine, however, that Tiffany commits some non-trifling but ultimately forgivable wrong. The recency of this wrongdoing may, per recency bias, increase its salience in Brett’s mind. Based on this temporary, unbalanced view of their relationship, Brett impetuously decides to break up and consumes an anti-love drug to prevent a future desire to undo this decision. In keeping with the subjective approach of this article to love and *akrasia*, let us assume that Brett would, on candid reflection,^16^ consider the decision to break up as an emotional and imprudent one, based on inferior reasons, and remaining together as the superior judgement. In this case, anti-love drugs would not serve their intended purpose of preventing akratic backsliding. Rather, they would permanently enshrine and preserve a normatively and subjectively undesirable decision.

Recognizing, then, that anti-love drugs are normatively desirable in some circumstances but not in others, it appears that their availability should be limited. The question of precisely *who* the gatekeeper of anti-love drugs’ availability should be is, however, a difficult one. Intimate relationships are deeply personal and, indeed, there may even be as many unique sets of circumstances as there are relationships. Furthermore, as I have said, a person’s complex motivations – and, particularly, whether a preference belongs to a higher or lower order – are largely opaque to outside parties. Though I intend to leave this question largely open, one possible, though admittedly imperfect, solution might be to make anti-love drugs available like other precommitment-based drugs, such as disulfiram and naltrexone, only on prescription by a licenced medical professional. I do not, of course, wish to valorize doctor-patient relationships or obscure academic criticism thereabout [51]. Nonetheless, this relationship is one which ought to be marked, at least, by elements of care, communication, knowledge, and trust [52–54]. As such, while no one is perfectly placed to act as the gatekeeper of anti-love drugs, the doctor-patient relationship might provide a useful basis to avoid their misuse and ensure they are only made available for their intended purpose of protecting against akratic love.

Given that the appointment of a doctor to act as a gatekeeper for anti-love drugs is an admittedly imperfect solution, it is prudent to remain open to alternatives. One such alternative – admittedly speculative in nature^17^—is the involvement of a so-called *enhancement counselor*, a role advocated by Emma Gordon in her recent book on human enhancement [55]. While the minutiae of Gordon’s work are beyond the scope of this article, she envisions the enhancement counselor as having a vital role in fostering and securing informed consent by those seeking to undergo voluntary enhancement.^18^ Most importantly for present purposes, the counselor facilitates the fulfillment of what Gordon calls the *authenticity-theoretic desideratum*, namely that an enhancement (or, in this case, the consumption of an anti-love drug) is in alignment with values that the subject would endorse on reflection. In particular, Gordon argues that the use of appropriate forms of questioning as part of the counselling process can enable the subject to “more reliably gauge whether [the] changes envisioned would be in line with the kinds of values they’d endorse” [55, p. 76]. Given, as I have said, that a person’s complex web of preferences is largely inscrutable to third parties, the role of the enhancement counselor in enabling the subject to better understand their own preferences and values may be therefore useful (either on its own or in concert with the input of a doctor) in ensuring that anti-love drugs are used only in cases of *bona fide* akratic love.

## Conclusion

The main purpose of this article has been to enliven ethical discourse around anti-love drugs by contextualizing it within existing discourse on precommitment. To this end, I have advanced the notion of so-called akratic love. In short, akratic love characterises the desire to return to a former partner (or, at least, some instances thereof) as a form of *akrasia*; though the person has formed a better judgement at *t*^*1*^ against remaining in a relationship, their willpower wavers at *t*^*2*^ and they are wont to act on this inferior *t*^*2*^ judgement by returning to their former partner. The notion of akratic love provides a useful theoretical conceptualization of the purpose of anti-love drugs. Insofar as these drugs propose to allow a person to manipulate the circumstances of their decision-making at *t*^*2*^ to prevent themselves from returning to the object of their akratic love, they are an unambiguous example of a precommitment strategy.

Having argued that there are no knockdown arguments against precommitment strategies in a general sense, I have, I believe, provided a firm ethical basis for the use of anti-love drugs in some circumstances (i.e. those of *bona fide* akratic love). Despite this, however, I have also acknowledged the potential for misuse of anti-love drugs, insofar as they might be consumed impetuously to prevent a return to a relationship which one’s subjective best judgement would endorse. The availability of these drugs ought therefore be limited, potentially by requiring a prescription from one’s doctor and/or counselling with a suitably-trained professional. Ultimately, this article is not intended to represent a conclusive last word on anti-love drugs *qua* precommitment strategy. Rather, I have aimed to highlight a valuable, novel framework for future directions in their ethical discussion.

## Footnotes

1 In contrast to Young’s [4] attention-grabbing definition, which I have cited here, Earp and Savulescu note that the effects of both love and anti-love drugs must be understood in context. These drugs, they say, “cannot completely override a person’s free will” [3]. Rather, “the most likely scenario for the foreseeable future…will be more or less powerful loading of the dice – not sorcery” [3]. I will, however, proceed under the assumption that future anti-love drugs will be at least reasonably effective in achieving their intended effect.

2 That precommitment involves the *self*-imposition of constraints requires some elaboration. Some forms of precommitment – most notably Ulysses pacts [15] – necessarily involve another party, who is charged with constraining the precommitting agent at the future point in accordance with their prior preference. A gambling self-exclusion agreement, for example, necessarily relies on the gambling operator and its staff to refuse service to the self-excluded gambler. Nonetheless, this refusal of service ought still to be considered to represent a self-imposed constraint insofar as it is authorized only by the prior consent of the precommitting agent. Absent this prior authorization, the intervention to, say, prevent a self-excluded gambler from gambling would be an unambiguous instance of paternalism, rather than precommitment [16].

3 In this article, I focus on the *voluntary* consumption of anti-love drugs for the purpose of precommitting oneself not to return to a former relationship. As an anonymous reviewer has pointed out, it is equally feasible that anti-love drugs could be administered *involuntarily* and paternalistically, *inter alia* in cases where a person’s feelings of love reach some threshold of objective harmfulness. We might imagine, for example, a person who is so consumed with feelings of love for a former partner that they become unable to attend to their own basic needs or, even, develop suicidal ideation. Alternatively, the involuntary administration of anti-love drugs may be appropriate in cases where a person has developed unrequited feelings of love—rising to the level of dangerous obsession—for a celebrity or some other uninterested party. Nonetheless, the ethics of coercive medical treatment are fraught and complex and, without prejudice to this obviously important area of ethical discourse, I will therefore leave aside here the involuntary administration of anti-love drugs in favor of my present focus on their voluntary consumption.

4 As an anonymous reviewer has astutely noted, it is in fact the *consumption* of an anti-love drug in the appropriate dosage which is a precommitment strategy, rather than the drug itself. Without prejudice to this point, I will refer here to anti-love drugs as a precommitment strategy as a term of convenience.

5 Research into anti-love drugs is not entirely the realm of science fiction, though it is in its nascent stages. One promising example of developments in the area is the use of propranolol in the treatment of adjustment disorder following romantic betrayal. See [21].

6 Earp & Savulescu [3] write that one problem with using, for example, antiandrogen drugs to dampen one’s sex drive is that the effect is indiscriminate; it diminishes one’s libido generally, rather than in relation to one person. Here I envision a future anti-love drug with an effect targeted to one person.

7 Though this article considers the use of anti-love drugs in relation to a relationship which has been dissolved, they may equally be used to soften the pain of a relationship which has never existed and never will, i.e. unrequited love. See [26].

8 Diamond writes that “ignoring [social] contexts produces a distorted account of human experience.” [23, p. 174].

9 Others have similarly characterized the desire to return as a lower-order preference and the desire to break up as high-order, albeit without making the connection with *akrasia*. See [3, 6].

10 A desire to return to a former partner might, of course, be framed as an addiction, though I will not pursue this notion here. See [40, 41].

11 As an anonymous reviewer has noted, it is feasible that future anti-love drugs may not operate in an *ex ante*, preference-shaping fashion but, rather, in an *ex post* manner, like disulfiram, by making their consumer feel physically unwell when near their former partner. This is, of course, a distinct possibility and one which generates ethical discourse in its own right. Nonetheless, in keeping with the definition of anti-love drugs which predominates in much of the relevant literature [3, 4], I have instead chosen to focus here on future drugs which propose to diminish or extinguish feelings of love in an *ex ante* manner. On this conception, there are theoretical similarities between anti-love drugs’ operation and that of naltrexone, which reduces cravings for and nullifies the effects of opioids. On the use of naltrexone as a precommitment strategy, see [42].

12 One way of conceptualizing this is to consider precluding the development of a *t*^*2*^ preference to return as an intrinsic aspect of the *t*^*1*^ preference to end the relationship.

13 It might be objected that akratic love is irrational, though I am unwilling to countenance this suggestion due to the subjective conception of love I have adopted. Love, as I outlined previously, may be good or bad, healthy or unhealthy and, most importantly here, rational or irrational.

14 Gerald Dworkin [34] makes a similar argument in relation to Ulysses’ pact with his sailors.

15 I adopt here the common political definition of anarchy as the absence of government or rulers, albeit to describe the absence of *self*-government and *self*-rule. While political anarchism might be normatively defensible, I believe that the absence of self-government (a crucial component of personal autonomy) is not. Indeed, even Robert Wolff, one of political anarchism’s most prominent proponents, wrote, “The responsible man is not…anarchic, for he does acknowledge himself bound by moral constraints” [50].

16 The preference-shaping effects of the anti-love drug would, of course, prevent such candid reflection. I mean here that Brett would *hypothetically* conclude, on candid reflection, that rekindling the relationship with Tiffany is the superior judgement and that breaking up is the impetuous and inferior judgement.

17 The speculative challenges posed by anti-love drugs might, of course, necessitate speculative solutions.

18 For methodological reasons which are not relevant here, Gordon does not consider anti-love drugs to fall within the category of human enhancement. Nonetheless, I consider her advocacy of the enhancement counselor role highly relevant for present purposes.

## Declarations

### Animal welfare

The article does not involve studies on animals.
